# Equilibrium points and their stability of COVID-19 in US

**DOI:** 10.1038/s41598-024-51729-w

**Published:** 2024-01-18

**Authors:** Xiaoxi Hu, Zixin Hu, Tao Xu, Kai Zhang, Henry H. Lu, Jinying Zhao, Eric Boerwinkle, Li Jin, Momiao Xiong

**Affiliations:** 1 State Key Laboratory of Genetic Engineering and Innovation Center of Genetics and Development, School of Life Sciences, Fudan University, Shanghai, China; 2 Artificial Intelligence Innovation and Incubation Institute, Fudan University, Shanghai, China; 3 Department of Epidemiology, University of Florida, Gainesville, FL 32611 USA; 4 Department of Environmental Health Sciences, School of Public Health, University at Albany, State University of New York, Rensselaer, NY 12144 USA; 5 BeiGene, Cambridge, MA 02141 USA; 6 School of Public Health, The University of Texas Health Science Center at Houston, Houston, TX 77030 USA; 7 Department of Biostatistics and Data Science, School of Public Health, The University of Texas Health Science Center at Houston, P.O. Box 20186, Houston, TX 77030 USA

**Keywords:** Statistics, Computational models, Epidemiology

## Abstract

This study aims to develop an advanced mathematic model and investigate when and how will the COVID-19 in the US be evolved to endemic. We employed a nonlinear ordinary differential equations-based model to simulate COVID-19 transmission dynamics, factoring in vaccination efforts. Multi-stability analysis was performed on daily new infection data from January 12, 2021 to December 12, 2022 across 50 states in the US. Key indices such as eigenvalues and the basic reproduction number were utilized to evaluate stability and investigate how the pandemic COVD-19 will evolve to endemic in the US. The transmissional, recovery, vaccination rates, vaccination effectiveness, eigenvalues and reproduction numbers (${R}_{0}$ and ${R}_{0}^{end}$) in the endemic equilibrium point were estimated. The stability attractor regions for these parameters were identified and ranked. Our multi-stability analysis revealed that while the endemic equilibrium points in the 50 states remain unstable, there is a significant trend towards stable endemicity in the US. The study's stability analysis, coupled with observed epidemiological waves in the US, suggested that the COVID-19 pandemic may not conclude with the virus's eradication. Nevertheless, the virus is gradually becoming endemic. Effectively strategizing vaccine distribution is pivotal for this transition.

## Introduction

Since the emergence of COVID-19 in Wuhan, China, in December 2019, the United States has encountered five distinct surges or waves of the disease. As of the present date, reported cases in the U.S. have reached a total of 101,094,670. Notably, on January 4, 2023, the 7-day average of new cases stood at 67,243, signifying a 16.2% increase from the previous week's average of 57,847^1^. This observed trend raises inquiries concerning the multifaceted reasons behind the recurrent waves in the United States and the potential for widespread vaccination to conclusively curtail the pandemic. Various driving forces contribute to these surges, encompassing the evolution of novel virus variants capable of eluding immune responses, declining natural immunity stemming from prior infections, relaxed adherence to or the inadequate implementation of non-pharmaceutical interventions (NPIs), and fluctuations in the effectiveness of vaccines over time^2^. An often underestimated yet pivotal factor is the inherent instability within the transmission dynamics of COVID-19. It is crucial to comprehend these causal elements and devise efficacious strategies to gain control over, and potentially terminate, the pandemic.

There is optimism that vaccination, natural immunity, and adaptable non-pharmaceutical interventions (NPIs) could pave the way for ending the spread of COVID-19 and a return to normalcy. However, the ongoing mutations and natural selection processes are producing new variants that not only increase the virus's replication and transmission but also enable it to evade the immune system. Such variants pose significant concerns due to their potential for heightened transmissibility and their ability to bypass immunity acquired from both vaccines and natural infections^3^.

The COVID-19 pandemic has been catastrophic. As of January 10, 2023, the global tally of cases and fatalities stood at 660,131,952 and 6,690,473, respectively^4^. It's vital to develop a mechanistic analytic model to examine the COVID-19 transmission dynamics. Such a model is key to periodically exploring the unstable patterns in COVID-19's spread, identifying the main drivers behind its multiple waves, and gaining a deeper understanding of the challenges in eradicating the virus. Epidemic compartment model is the most commonly employed mathematical framework for analyzing COVID-19's transmission dynamics^5–11^. For instance, the Susceptible-Infected-Recovered (SIR) model, a staple in prior research, has been instrumental in simulating COVID-19 spread within communities^12–14^. Researchers have expanded the model to include an 'Exposed' (E) compartment, thereby evolving it into the SEIR model for a more accurate representation of the disease's progression^15–19^. However, since December 2020, the USFDA's emergency authorization of the Pfizer and Moderna mRNA vaccines marked a turning point^20^. With Phase 3 clinical trials indicating approximately 95% efficacy for both vaccines in preventing COVID-19^21^, governments and communities have been actively promoting vaccination. By the end of 2021, around 73% of the U.S. population had received at least one vaccine dose, with 62% fully vaccinated^22^, profoundly affecting the virus's transmission dynamics. This significant shift highlights the urgency to incorporate vaccination data into epidemic models^23,24^. On the other hand, the traditional mathematical models rely on a set of differential equations, merely estimating parameters from real data through numerical solutions of these equations falls short in fully capturing the dynamic nature of COVID-19 transmission and in formulating effective strategies to combat the virus. Stability analysis, using real-world data and often neglected in COVID-19 epidemic models, is crucial for understanding the virus's dynamic behavior. COVID-19's tendency to oscillate between stationary and non-stationary states leads to multiple epidemic waves, a complex pattern that demands thorough analysis.

To tackle these challenges, we propose an augmentation to the SEIR model by integrating a vaccination component, resulting in the SEIRV model. Our primary objective involves conducting comprehensive stability analyses of the SEIRV model within the context of COVID-19^10,11,22,25–27^. We intend to delineate both the disease-free and epidemic critical equilibrium points and subsequently employ advanced next-generation matrix methods to compute the basic reproduction numbers pertaining to these critical points: ${R}_{0}$ for the disease-free state and ${R}_{0}^{end}$ for the epidemic state. Employing stability theory, we aim to derive conditions conducive to stable states within the SEIRV system. Moreover, our endeavor includes the identification of attractors defining the spectrum of parameters governing stable states within the dynamic system of COVID-19^28^. Leveraging this multifaceted stability analysis, our overarching aim is to provide critical insights to aid in devising strategies aimed at mitigating and ultimately arresting the transmission of COVID-19 in the United States.

## Methods

### Data sources

The number of cases and deaths for each state from January 21, 2020 to December 12, 2022 was downloaded from https://github.com/nytimes/covid-19-data. The vaccination data, including the number of vaccines distributed, the number of people who received at least one shot of vaccinations and full vaccination for each state from January 12, 2021 to December 12, 2022 were downloaded from https://ourworldindata.org/us-states-vaccinations.

### SEIRV compartment model for COVID-19 transmission dynamics

We constructed a compartmental model based on a deterministic system of nonlinear differential equations for COVID-19 transmission dynamics, which further took into account vaccination. The dynamical system was established based on the assumptions:

1. We assumed that the total population could be classified into several compartments, and the transition of individuals from one compartment to another depends on the stage of the disease;
2. We assumed that if susceptible individuals exposed to an infectious source, they are not infectious until they have symptoms after a latent period;
3. We assumed that the infected individuals will recover or die after an infectious period, however, for model simplicity we put them into one group which is called recovered;
4. We assumed a constant recruiting rate and natural death rate.

Based on these assumptions, we established the model including the susceptible ($S(t)$), exposed ($E(t)$), infected ($I(t)$), recovered ($R(t)$), and vaccinated ($V(t)$) individuals, each one forming a compartment. The model employed a constant recruiting rate (births) $\Lambda$ to the susceptible individuals ($S(t)$), natural death rate $\mu$, transmission rate $\beta ,$ vaccination rate $\alpha$, incubation rate $\gamma$, the probability of the recovery or death $\delta$, and vaccine inefficiency $\sigma$. The detailed definition of these populations and description of the transmission relationship among them have been concluded in Table 1.

**Table 1:** Description of notations and parameters in the model, the first column denotes the symbols and the second one denotes the description.

Compartments	
$S(t)$	Susceptible individuals, the individuals who are not infected, but may become infected in the future
$E(t)$	Exposed individuals, the individuals who are already infected, but not yet infectious
$I(t)$	Infected individuals, the individuals who have already been infected and can transmit it to the susceptible individuals
$V(t)$	Vaccinated individuals, the individuals who are vaccinated, but still have the possibility to be infected
$R(t)$	Recovered individuals, the individuals who have recovered from the virus and are assumed to be not infected any more
N	The total population size, $N=S\left(t\right)+E\left(t\right)+I(t)+R\left(t\right)+V(t)$
Parameters	
$\Lambda$	Recruiting rate (births), $\Lambda = \mu N$
$\mu$	Natural death rate
$\beta$	Transmission rate, the rate at susceptible compartment transmit to exposed one; it has been divided by the total population N for calculation
$\alpha$	Vaccination rate, the rate at susceptible individuals become vaccinated
$\gamma$	Incubation rate, the rate at exposed population moves to infected one
$\delta$	The probability of the recovery or death due to COVID-19, the rate at infected persons become recovered or dead caused by COVID-19
$\sigma$	The vaccine inefficiency, the rate at vaccinated people become exposed, $1-\sigma$ represents the population vaccine efficacy

**Figure 1:**
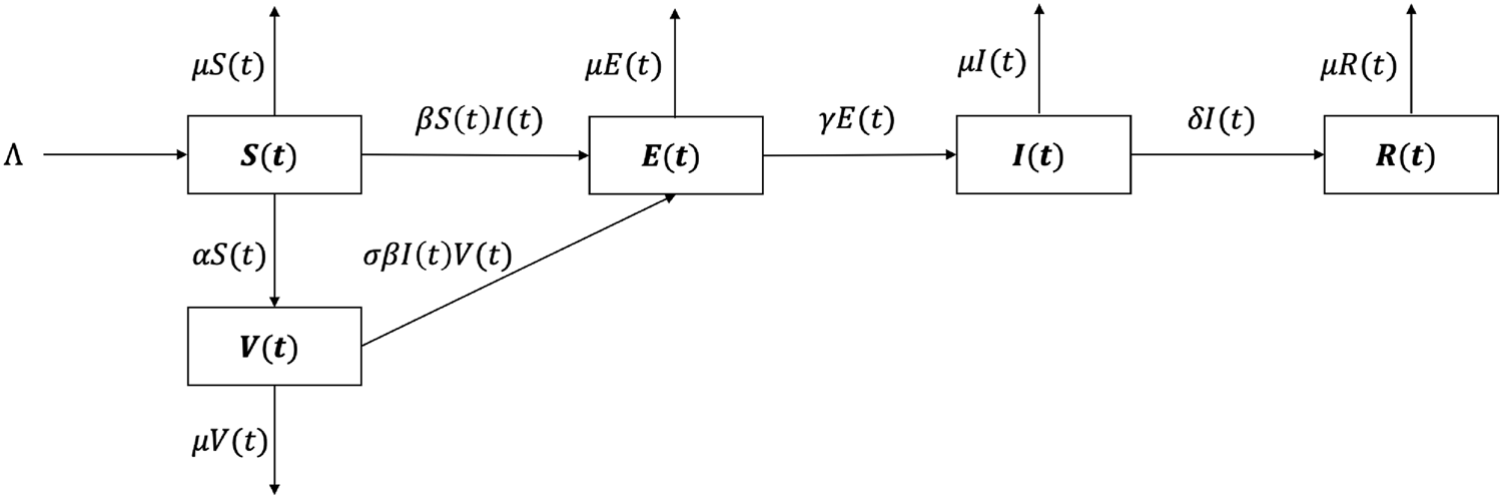
Flow diagram of the dynamic transmission model of COVID-19.

We plotted Fig. 1 to depict the transitional relationships among the various states within the model. Based on the above assumptions and concepts, the rates of change of the five populations were governed by the following system of nonlinear ordinary differential equations, which constituted the SEIRV model used in our study:

1 $$\frac{dS(t)}{dt}=\Lambda -\beta S\left(t\right)I\left(t\right)-\alpha S\left(t\right)-\mu S\left(t\right),$$

2 $$\frac{dE(t)}{dt}=\beta S\left(t\right)I\left(t\right)-\gamma E\left(t\right)+\sigma \beta I\left(t\right)V\left(t\right)-\mu E\left(t\right),$$

3 $$\frac{dI(t)}{dt}=\gamma E\left(t\right)-\delta I\left(t\right)-\mu I\left(t\right),$$

4 $$\frac{dR(t)}{dt}=\delta I\left(t\right)-\mu R\left(t\right),$$

5 $$\frac{dV(t)}{dt}=\alpha S\left(t\right)-\sigma \beta I\left(t\right)V\left(t\right)-\mu V\left(t\right).$$

Summarizing the above equations, we obtained

6 $$\frac{dN}{dt}=\Lambda -\mu N\left(t\right).$$

From Eq. (6), we can rewrite $N\left(t\right)$ as

7 $$N\left(t\right)=\frac{\Lambda }{\mu }+\left(N\left(0\right)-\frac{\Lambda }{\mu }\right){e}^{-\mu t}.$$

We assumed that $N\left(0\right)\le \frac{\Lambda }{\mu },$ so we obtain $N(t)$ is bounded by $\frac{\Lambda }{\mu }$ for all $t$,

8 $$N\left(t\right)\le \frac{\Lambda }{\mu }.$$

Thus, we showed that all variables $S\left(t\right), E\left(t\right), I\left(t\right), R\left(t\right), V(t)$ were bounded in the region $\Omega$, and more details could be found in Supplementary Note S2:

9 $$\Omega =\left\{{N}\left({t}\right)={S}\left({t}\right)+{E}\left({t}\right)+{I}\left({t}\right)+{R}\left({t}\right)+{V}\left({t}\right)\right|0\le N(t)\le \frac{\Lambda }{\mu }\}.$$

### Reproduction number

The basic reproduction number, denoted as ${R}_{0}$, is an important threshold quantity which determines whether the COVID-19 will continuously spread in the population or disappear. It is defined as the average number of secondary cases produced by one infected individual introduced into a population of susceptible individuals^29^.

For the set containing all infected individuals (${E}\left({t}\right)$ and ${I}\left({t}\right)$), we defined

10 $$X\left(t\right)=\left[\begin{array}{c}E(t)\\ I(t)\end{array}\right].$$

There is

11 $$\begin{gathered} \frac{dX}{{dt}} = \left[ {\begin{array}{*{20}c} {\frac{dE}{{dt}}} \\ {\frac{dI}{{dt}}} \\ \end{array} } \right] \hfill \\ = \left[ {\begin{array}{*{20}c} { - \left( {\gamma + \mu } \right)E\left( t \right) + \beta \left( {S\left( t \right) + \sigma V\left( t \right)} \right)I\left( t \right)} \\ {\gamma E\left( t \right) - \left( {\delta + \mu } \right)I\left( t \right)} \\ \end{array} } \right] \hfill \\ = \left[ {\begin{array}{*{20}c} {\beta \left( {S\left( t \right) + \sigma V\left( t \right)} \right)I\left( t \right)} \\ 0 \\ \end{array} } \right] - \left[ {\begin{array}{*{20}c} {\left( {\gamma + \mu } \right)E\left( t \right)} \\ { - \gamma E\left( t \right) + \left( {\delta + \mu } \right)I\left( t \right)} \\ \end{array} } \right] \hfill \\ \mathop = \limits^{{{\text{def}}}} {F}\left( X \right) - {V}\left( X \right) \hfill \\ \end{gathered}$$

Let

12 $$F'\left(X\right)=\left[\frac{\partial F\left(X\right)}{\partial {X}^{T}}\right]=\left[\begin{array}{cc}0& \beta \left(S\left(t\right)+\sigma V\left(t\right)\right)\\ 0& 0\end{array}\right]$$

13 $$V'\left(X\right)=\left[\frac{\partial V(X)}{\partial {X}^{T}}\right]=\left[\begin{array}{cc}\left(\gamma +\mu \right)& 0\\ -\gamma & (\delta +\mu )\end{array}\right].$$

The basic reproduction number ${R}_{0}$ was defined as the spectral radius of the next-generation matrix $F'\left(X\right){V'}^{-1}\left(X\right)$ as the following, and more detailed calculation was illustrated in Supplementary Note S3.1^29^:

14 $${R}_{0}=\frac{\gamma \beta \left(S\left(t\right)+\sigma V\left(t\right)\right)}{\left(\gamma +\mu \right)(\delta +\mu )}.$$

The reproduction number ${R}_{0}$ is used to measure the transmission potential of a disease. Intuitively, we can expect that if ${R}_{0}<1$ then the number of new cases of COVID-19 will decrease, and the number of new cases will increase if ${R}_{0}>1$.

### Steady sate analysis of SEIRV for COVID-19 transmission dynamic system

Predictions of multistable structural dynamics are paramount to the development and implementation of vaccination and population public health intervention for controlling the spread of COVID-19 under highly uncertain biological, economic, political, and environmental perturbation. Although a direct numerical solution to differential equations can be used to calculate the steady state, analytic analysis of the steady state can reveal how the parameters affect the steady state and enable prediction of near- and far- from equilibrium response. Such analyses are invaluable for understanding the endemic waves of COVID-19 and developing strategies to mitigate or even end the pandemic.

COVID-19 transmission dynamic system is an autonomous system in which the independent time variable $t$ does not explicitly appear in the differential equations. We will focus on steady state and stability analysis of autonomous systems to investigate the qualitative dynamic behavior of COVID-19 dynamic systems. The central issue in stability analysis is to identify isolated critical (equilibrium) points of COVID-19 transmission dynamics. The isolated critical points can be classified as disease-free (number of new cases is zero) critical point and endemic (new cases are not zero) critical point. The two classes of critical points are given as follows (For details, please see Supplementary Note S3.2).

(1) Disease-free critical (equilibrium) point:

15 $${I}_{*}=0, {{E}_{*}=0, R}_{*}=0, {S}_{*}=\frac{\Lambda }{\alpha +\mu }, {V}_{*}=\frac{\alpha\Lambda }{\mu (\alpha +\mu )},{R}_{0}=\frac{\gamma \beta\Lambda \left(\mu +\alpha \sigma \right)}{\mu \left(\gamma +\mu \right)(\delta +\mu )(\alpha +\mu )}$$

(2) Endemic critical (equilibrium) point:

16 $$\begin{gathered} I_{*} = \frac{{ - b + \sqrt {b^{2} - 4ac} }}{2a}, \hfill \\ a = \frac{{\left( {\gamma + \mu } \right)\left( {\delta + \mu } \right)\sigma \beta^{2} }}{\gamma },\,\,\,b = \frac{{\left( {\gamma + \mu } \right)\left( {\delta + \mu } \right)\left[ {\mu \beta + \left( {\alpha + \mu } \right)\sigma \beta } \right]}}{\gamma } - \sigma \beta^{2} {\Lambda }, \hfill \\ c = \frac{{\mu \left( {\gamma + \mu } \right)\left( {\delta + \mu } \right)\left( {\alpha + \mu } \right)}}{\gamma } - \beta {\Lambda }\left( {\mu + \alpha \sigma } \right), \hfill \\ S_{*} = \frac{{\Lambda }}{{\alpha + \mu + \beta I_{*} }},{ }V_{*} = \frac{{\alpha {\Lambda }}}{{\left( {\mu + \sigma \beta I_{*} } \right)\left( {\alpha + \mu + \beta I_{*} } \right)}},{ }E_{*} = \frac{\delta + \mu }{\gamma }I_{*} ,{ }R_{*} = \frac{\delta }{\mu }I_{*} , \hfill \\ R_{0}^{end} = \frac{{\gamma \beta {\Lambda }}}{{\left( {\gamma + \mu } \right)\left( {\delta + \mu } \right)\left( {\alpha + \mu + \beta I_{*} } \right)}}\left[ {1 + \frac{\alpha \sigma }{{\left( {\mu + \sigma \beta I_{*} } \right)}}} \right] \hfill \\ \end{gathered}$$

### Classification of critical points

Stability analysis for the general nonlinear dynamic systems is complicated. In this paper, we will focus on isolated critical point and almost linear systems. A system is called almost linear at a critical point if the Jacobian matrix of linearized system at an isolated critical point is invertible. We assumed Jacobian matrix of the nonlinear dynamic system (1–5) is invertible, and could be derived as

17 $${J}_{*}=\left[\begin{array}{ccc}-{\varepsilon }_{1}& 0& \begin{array}{ccc}-\beta {S}_{*}& 0& 0\end{array}\\ \beta {I}_{*}& -{\varepsilon }_{2}& \begin{array}{ccc}\beta ({S}_{*}+\sigma {V}_{*})& 0& \sigma \beta {I}_{*}\end{array}\\ \begin{array}{c}0\\ 0\\ \alpha \end{array}& \begin{array}{c}\gamma \\ 0\\ 0\end{array}& \begin{array}{ccc}\begin{array}{c}-{\varepsilon }_{3}\\ \delta \\ -\sigma \beta {V}_{*}\end{array}& \begin{array}{c}0\\ -\mu \\ 0\end{array}& \begin{array}{c}0\\ 0\\ -{\varepsilon }_{4}\end{array}\end{array}\end{array}\right],$$

where

18 $$\varepsilon_{1} = \alpha + \mu + \beta I_{*} ,{ }\varepsilon_{2} = \gamma + \mu ,{ }\varepsilon_{3} = \delta + \mu \,\,{\text{and}}\,\,\varepsilon_{4} = \mu + \sigma \beta I_{*}$$

Its characteristic polynomial is

19 $$\begin{aligned} \left| {\lambda I_{*} - J_{*} } \right| =& \left( {\lambda + \mu } \right)\{ \left( {\lambda + \varepsilon_{4} } \right)[\left( {\lambda + \varepsilon_{1} } \right)\left( {\lambda + \varepsilon_{2} } \right)\left( {\lambda + \varepsilon_{3} } \right) \hfill \\ &+ \gamma \beta [\beta I_{*} S_{*} - \left( {\lambda + \varepsilon_{1} } \right)\left( {\left( {S_{*} + \sigma V_{*} } \right)} \right] + \gamma \beta^{2} \left[ {\left( {\lambda + \varepsilon_{1} } \right)\sigma^{2} I_{*} V_{*} + \alpha \sigma I_{*} S_{*} } \right]\} , \hfill \\ \end{aligned}$$

where $\lambda$ are the corresponding eigenvalues.

Using Routh–Hurwitz stability criterion, we could obtain the positivity or negativity of the eigenvalues, which could help us study the properties of the critical points which determine whether the system is stable or unstable or whether the number of new cases decreases or increases. Disease-free and endemic critical points are separately investigated and briefly presented (for details, please see Supplementary Note S3.3).

- **Disease-free critical (equilibrium) point**

Let $${R}_{0}=\frac{\gamma \beta\Lambda \left(\mu +\alpha \sigma \right)}{\mu \left(\gamma +\mu \right)(\delta +\mu )(\alpha +\mu )}$$.

The disease-free critical point can be classified as three cases:

1. when ${R}_{0}<1$, the disease-free critical point is classified as an asymptotically stable node;
2. when ${R}_{0}=1$, the disease-free critical point is classified as an unstable node;
3. when ${R}_{0}>1$, the disease-free critical point is classified as an unstable saddle point.

- **Endemic critical (equilibrium) point**

Let $${R}_{0}^{end}=\frac{\gamma \beta\Lambda }{(\gamma +\mu )(\delta +\mu )(\alpha +\mu +\beta {I}_{*})}\left[1+\frac{\alpha \sigma }{(\mu +\sigma \beta {I}_{*})}\right]$$.

Then, in summary, the endemic equilibrium point can be classified into three cases:

1. when ${R}_{0}^{end}<1$, the endemic equilibrium point is classified as an asymptotically stable node;
2. when ${R}_{0}^{end}=1$, the endemic equilibrium point is classified as an unstable node;
3. when ${R}_{0}^{end}>1$, the endemic equilibrium point is classified as an unstable saddle point.

### Parameter estimation

We can use the steady states of the COVID-19 transmission dynamic system to estimate the parameters in the nonlinear differential equation models and then use the estimated parameters to identify and classify the equilibrium points.

Let

20 $$\begin{gathered} a = \frac{{\sigma \left( {\gamma + \mu } \right)\left( {\delta + \mu } \right)\beta^{2} }}{\mu } \hfill \\ b = \frac{{\left( {\gamma + \mu } \right)\left( {\delta + \mu } \right)\beta }}{\gamma \mu }\left[ {\mu + \left( {\alpha + \mu } \right)\sigma } \right] - \frac{{\Lambda \sigma \beta^{2} }}{\mu } \hfill \\ c = \frac{{\left( {\gamma + \mu } \right)\left( {\delta + \mu } \right)\left( {\alpha + \mu } \right)}}{\gamma } + \beta \left[ {\Lambda \sigma - \Lambda - \frac{\alpha + \mu }{\mu }\Lambda } \right] \hfill \\ d = \left( {\Lambda - \mu } \right)\left( {\alpha + \mu } \right) \hfill \\ F\left( {\Lambda , \mu ,\delta , \gamma , \beta , \alpha , \sigma } \right) = aI^{3} + bI^{2} + cI + d. \hfill \\ \end{gathered}$$

The parameters were estimated by minimizing

21 $$\underset{\Lambda ,\upmu ,\updelta ,\upgamma ,\upbeta , \alpha ,\upsigma }{{\text{min}}}{F\left(\Lambda ,\upmu ,\updelta ,\upgamma ,\upbeta , \alpha ,\upsigma \right)}^{2},$$

where $I$ is the observed number of new cases in the steady state (hypothesized) of COVID-19 dynamic system (1–5).

### Ethical approval

This study did not involve individual information, so there was no requirement for informed consent.

## Real data analysis

### Estimation of parameters

**Figure 2:**
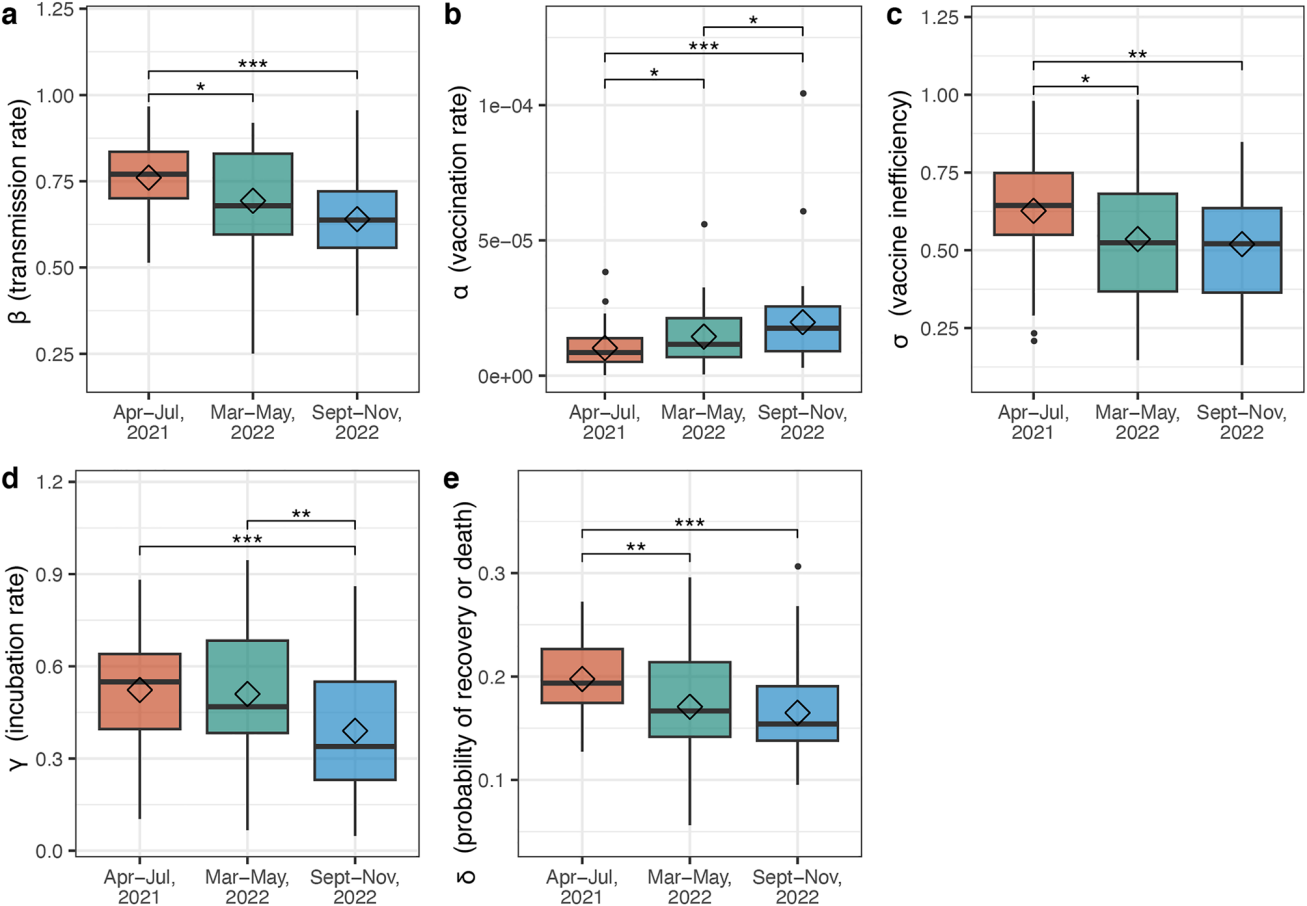
Box plot for the parameters $\beta , \alpha ,\sigma , \gamma , \delta$ in three steady periods (April–July, 2021; March–May 2022; September–November, 2022) across 50 states in the US. (**a**) Box plot for the transmission rate $\beta$, representing the rate at susceptible compartment transmit to exposed one, (**b**) box plot for the vaccination rate $\alpha$, representing the rate at susceptible individuals become vaccinated, (**c**) box plot for the vaccine inefficiency $\sigma$, representing the rate at vaccinated people become exposed, (**d**) box plot for the incubation rate $\gamma$, representing the rate at exposed population moves to infected one and (**e**) box plot for the probability of the recovery or death $\delta$, representing the rate at infected persons become recovered.

The data we employed started from January 12, 2021, and ended at December 12, 2022. To examine endemic equilibrium points, we identified three distinct steady state periods: (1) April–July, 2021; (2) March–May, 2022; and (3) September–November, 2022 (Fig. S1). The daily reported new infection cases were utilized to calibrate the COVID-19 model, enabling precise estimation of the model parameters. The parameters are estimated by minimizing the Eq. (21). We applied the black-hole optimizer (BHO) approach, which is considered as a recent metaheuristic optimization technique and is used to solve continuous optimization tasks by adapting physical behaviors in evolution rules^30–33^. For all the states, we assumed a same and constant birth rate $\frac{\Lambda }{N}$ and natural death rate $\mu$. The other five parameters $\alpha ,\beta , \gamma , \delta ,$ and $\sigma$ are derived for each state individually using their respective observed number of new cases and total population size. In order to avoid the impact of random initial setting on the results, for each state, we performed estimation of 100 times and selected the parameters that minimized the loss of Eq. (21). Within each estimation process, the maximum number of iterations was set as 5000. Figure 2 displayed the estimated median values and standard deviations of the parameters $\alpha ,\beta , \gamma , \delta ,$ and $\sigma$ across three steady periods for all 50 states in the US. We observed that comparing these three steady state periods, the trends for parameters $\beta , \sigma , \gamma ,$ and $\delta$ showed a decreasing pattern, with only parameter $\alpha$ increasing. For all parameters, the changes from the time periods April–July, 2021 to September–November, 2022 were significant using paired wilcoxon signed-rank test ($\beta :p<0.0001$, $\sigma :p=0.0021$, $\gamma :p=0.0008$, $\delta :p<0.0001$, $\alpha :p=0.0003$). The decreases in both transmission rate $\beta$ and incubation rate $\gamma$ indicated a significant shift in the dynamics of the disease spread, suggesting that the disease was spreading more slowly, likely due to effective public health interventions. Meanwhile, the decrease in vaccine inefficacy rate $\sigma$ coupled with an increase in vaccination rate $\alpha$ reflected a positive development in vaccine-related defenses. Vaccines became more effective and more widely administered, boosting population immunity. In summary, the changes observed in the parameters suggested a gradual reduction in COVID-19 spread rates in the US, likely attributable to the escalating vaccination efforts.

### Eigenvalues, reproduction number at the endemic equilibrium points and stability analysis

To evaluate the stability of COVID-19's dynamics across the 50 states in the US, we analyzed the eigenvalues of the Jacobian matrix at endemic equilibrium points. Eigenvalues could help determine the stability of the virus’ spread, assessing whether the virus's spread will be killed or persist within a population. Our system had a total of five eigenvalues, with theoretical stability analysis revealing that one was consistently negative. Notably, in all three steady periods, our calculations revealed that at least one eigenvalue was positive in each state (Table S1), indicating that the COVID-19 dynamics across the US have not yet stabilized. However, there was a notable decrease in the largest eigenvalue in 32 states from the first (April–July, 2021) to the third steady period (September–November, 2022), while 3 states exhibited no significant changes during these periods.

**Figure 3:**
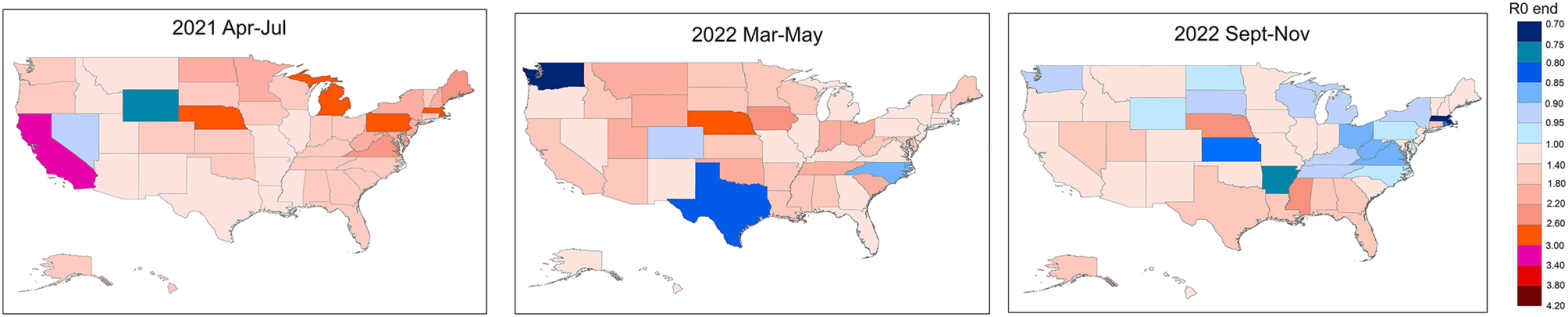
Map of distributions of the reproduction number ${R}_{0}^{end}$ in three steady periods across 50 states in the US. This figure was generated using ArcGIS 10.6 (ESRI, Redlands, California USA; URL: http://www.esri.com/).

The reproduction number ${R}_{0}^{end}$ at the endemic equilibrium point indicates the average number of people that one infected individual will infect in a fully susceptible population, and can be used to characterize how far the dynamic system is from the stable state. Values of ${R}_{0}^{end}$ exceeding 1 suggest a potential epidemic, while values below 1 indicate a possible decline in the disease; if they stabilize around 1, COVID-19 may become endemic, maintaining predictable levels within the population. Table S2 presented the reproduction number ${R}_{0}^{end}$ for all three steady periods across 50 states in the US, revealing that the number of states with ${R}_{0}^{end}$ less than 1 were 2, 4, and 19 in the first, second and third steady period, respectively. Figure 3 featured a map showing the distribution of ${R}_{0}^{end}$ values across these periods in the US. Notably, from the result of the third steady period, most states in the Middle East were expected to reach a steady state in the latest COVID-19 wave in the US. A comparison between Tables S1 and S2 indicated that 16 states with an ${R}_{0}^{end}$ below 1 in the third steady period corresponded to those showing a decrease in the largest eigenvalues from the second to the third period.

### Impact of the parameters on the eigenvalues

**Figure 4:**
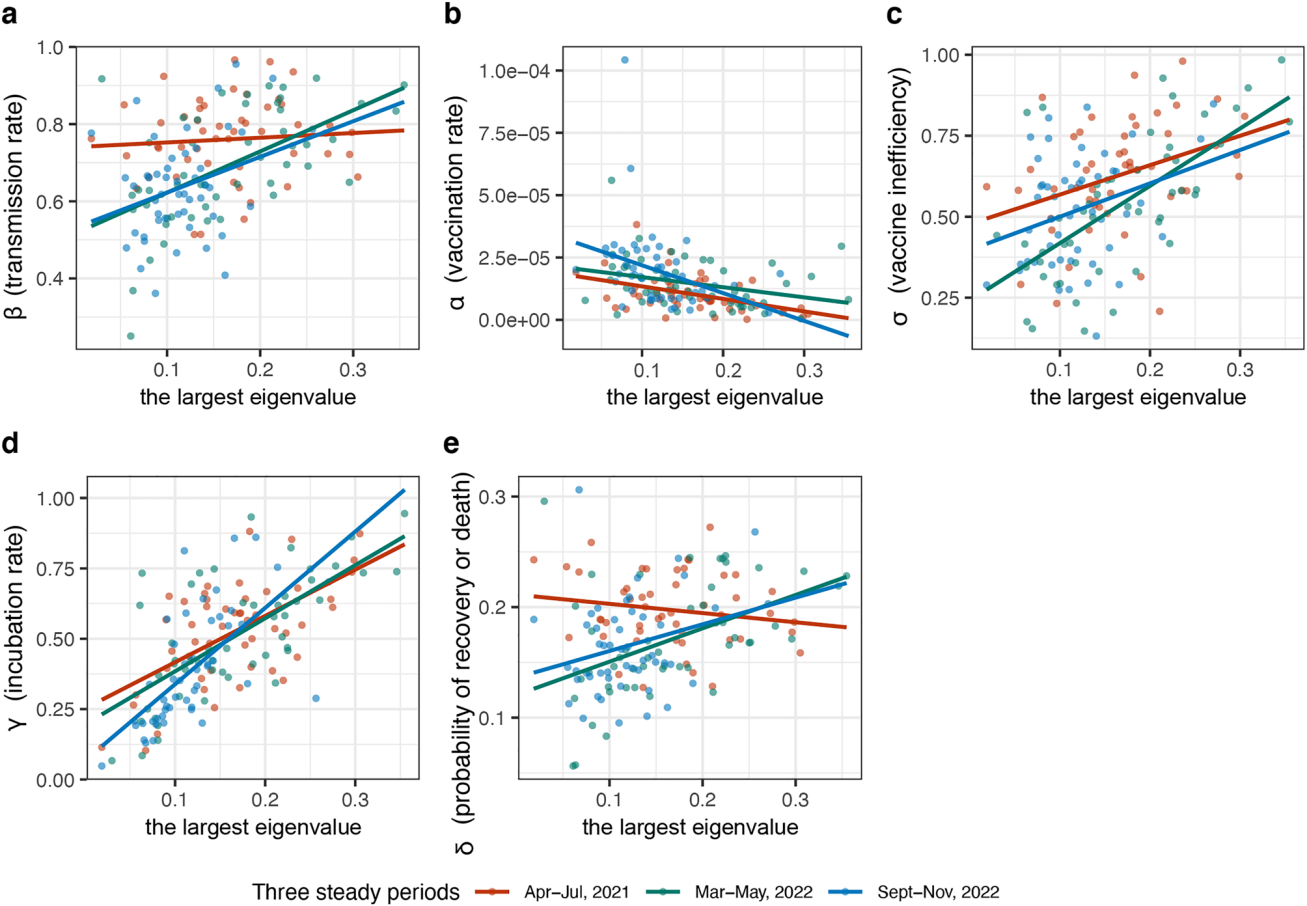
The relationship between the parameters and the largest eigenvalue of the COVID-19 dynamic systems. (**a**) The relationship between transmission rate $\beta$ and the largest eigenvalue, (**b**) the relationship between vaccination rate $\alpha$ and the largest eigenvalue, (**c**) the relationship between vaccine inefficiency $\sigma$ and the largest eigenvalue, (**d**) the relationship between incubation rate $\gamma$ and the largest eigenvalue, and (**e**) the relationship between probability of the recovery or death $\delta$ and the largest eigenvalue.

To illustrate the impact of the parameters $\beta , \alpha , \sigma , \gamma ,$ and $\delta$ on the largest eigenvalue of the COVID-19 dynamics, we plotted Fig. 4 to demonstrate the relationship between these parameters and the largest eigenvalue in the COVID-19 dynamic systems. Our observations revealed a positive correlation of the largest eigenvalue with $\beta$, $\sigma$, $\gamma$ in all three steady periods, and with δ in the second and third periods. In contrast, there was a negative correlation with the parameter $\alpha$. Therefore, to lower the largest eigenvalue, and thereby the disease's spread, it's necessary to decrease the rates of transmission $\beta$, reduce the time individuals that spent in infectious state $\gamma$, increase the recovery rate $\delta$, increase the vaccination rate $\alpha$, and improve vaccine effectiveness $1-\sigma$. This combination of strategies would collectively reduce the spread of the COVID-19 within the population.

### Approximate attractor analysis and impact of the parameters on stability

An attractor is defined as a set of states toward which a system attempts to return after perturbation^34^. Rather than examining the attractors of the state variables $I, V, S, E$, and $R$ in our system, we focused on identifying the parameter regions that govern the stability of COVID-19 dynamics and the effects of parameter variations on this stability. Our model incorporated five estimated parameters, presenting a challenge in simultaneously depicting and visualizing the stability region encompassing all these parameters.

Since the vaccination rate $\alpha$ and vaccine inefficiency rate $\sigma$ jointly contribute to the transmission dynamics of COVID-19, we investigated the impact of parameters $\alpha$ and $\sigma$ together on the stability of COVID-19 dynamics. Figure S2 showed the impact of simultaneously varying parameters $\alpha$ and $\sigma$ while maintaining the current values of other parameters unchanged on the stability of COVID-19 dynamics in the third steady period of September–November 2022 across 50 states in the US. In this figure, the stability region of the parameters was represented in blue color, while the instability region was shown in red color. An upward movement along the vertical axis represented an increase in the vaccination rate $\alpha$, and a leftward movement on the horizontal axis signified a decrease in the vaccine inefficiency rate $\sigma$ or, conversely, an increase in vaccine efficacy. Our observations from Fig. S2 indicated that Arkansas (AR), Kentucky (KY), Idaho (ID), and Georgia (GA) had the largest stability regions for $\alpha$ and $\sigma$, whereas Alaska (AK), Florida (FL), Louisiana (LA), Connecticut (CT), and Texas (TX) had the smallest. The above finding offered crucial insights that enhancing vaccination rates can compensate for low vaccine efficiency, and similarly, improving vaccine efficiency can balance a lower vaccination rate to maintain stability.

Subsequently, we examined the combined effect of parameters $\gamma$ (incubation rate) and $\delta$ (probability of recovery or death) on the stability of COVID-19 dynamics. Figure S3 displayed the impact of simultaneously changing $\gamma$ and $\delta$, while holding the values of other parameters constant, on the stability of COVID-19 dynamics during the third steady period. Our analysis indicated that increasing $\delta$ and decreasing $\gamma$ would nudge the COVID-19 dynamic system closer to stability. In a similar vein, Figs. S4 and S5 demonstrated the consequences of changing parameters $\beta$ and $\gamma$, and $\beta$ and $\delta$, respectively, under the same conditions, on the stability of COVID-19 dynamics across the states. Furthermore, Fig. S6 illustrated the effects of altering $\beta , \gamma ,$ and $\delta$ simultaneously, while keeping other parameters constant, on the stability regions in selected states. It specifically represented states with the largest and second largest stability regions, Michigan (MI) and Kansas (KS), against those with the smallest and second smallest stability regions, Nebraska (NE) and Washington (WA). In summary, these figures showed that implementing strategies decreasing transmission rate $\beta$, decreasing incubation rate $\gamma$, and increasing the probability of recovery or death $\delta$ simultaneously, or any two of them, would drive the dynamic system of COVID-19 toward stability region. Biologically, decreasing $\beta$ meant fewer individuals are infected over time, lowering the spread of the virus. Reducing $\gamma$ would extend the time before exposed individuals can infect others, reduce the rate of infection, and provide a chance to contain the virus through early intervention, such as isolation or treatment. Increasing $\delta$ would lead to quicker recuperation, shrinking the pool of infectious individuals. When these changes occur together, or even just two of them, it would slow the disease transmission and guide COVID-19 dynamics toward stability.

## Conclusions

At present, the most pressing and widespread question concerns the end of the COVID-19 pandemic^35^. While some scientists have optimistically forecasted the pandemic's conclusion in 2022^36^, others caution that it is far from over^37–39^. In response to this debate, our paper focused on developing a method to analyze the stability of COVID-19 transmission dynamics. This approach serves as a tool to evaluate whether the pandemic is transitioning into an endemic phase.

There is no universally agreed-upon definition of a pandemic^40,41^. Pandemic and endemic are terms rooted in systems dynamics. In our study, we interpreted “endemic” as the “stable states” of the COVID-19 dynamic system. Generally, protection against COVID-19 infection relies on vaccination and natural immunity, which of both are important to be considered in modelling, but the data for natural immunity is missing. In this paper, we proposed an innovative approach, which embodied in the integration of vaccination into the SEIR model to study the stability of the COVID-19 dynamics. By accounting for vaccinated individuals as a separate compartment, we could capture the nuanced impacts of vaccination campaigns on disease dynamics, offering insights into anticipate the effects of vaccine rollouts on the transmission of the virus. This advancement not only enhances the model's ability to simulate real-world scenarios, but also provides a valuable tool for public health planners to optimize vaccination programs and mitigate the spread of the disease. Specifically, we formulated an objective function to ensure the steady states of dynamics which was then applied in estimating the parameters driving the current transmission dynamics of COVID-19. We derived eigenequations to determine eigenvalues, as well as reproduction number ${R}_{0}$ at disease-free equilibrium point and ${R}_{0}^{end}$ at the endemic equilibrium point, to assess the stability of COVID-19 dynamics under these conditions. Our analysis led to two significant conclusions. The first was that all 50 states in the US were in unstable states during all three steady periods. The second conclusion, however, indicated that the COVID-19 dynamics are moving towards stability. Analysis of real data revealed that the three transition-related parameters $\beta , \gamma$ and $\delta$ decreased while the vaccination rate $\alpha$ and vaccine efficiency $1-\sigma$ increased, progressing from the first steady period through the second one to the third period. Again, the results also showed that both the largest eigenvalue of the stability analysis matrix and the reproduction number ${R}_{0}^{end}$ in three steady periods decreased consistently across the three steady periods. Both vaccination and non-pharmaceutical interventions (NPIs) are critical in curbing the spread of COVID-19. Theoretically, by exploring the parameter space of vaccination-related factors $\alpha , \sigma$ and NPI-related factors $\beta , \gamma , \delta$, we can define the stability region of these parameters during steady state periods. In this paper, we have calculated the stability regions for $\alpha , \sigma$, and $\beta , \gamma , \delta$, offering insights into transitioning COVID-19 from a pandemic to an endemic state. Enhancing vaccine efficacy, such as through the use of mucosal vaccines, coupled with an increased vaccination rate and the implementation of appropriate NPIs, can facilitate this change from pandemic to endemic.

In conclusion, our research established a novel SEIRV model and thus provided compelling evidence that COVID-19 will not conclude with the virus's elimination but is transitioning towards an endemic stage in the United States. The stability analysis, aligned with observed epidemic patterns, underscored this inevitable shift. Critically, our findings emphasized the importance of strategically designed vaccine deployment as a determinant in this evolution. Effective vaccination strategies are pivotal not only in mitigating the immediate impact of the pandemic but also in shaping the long-term coexistence with the virus. This research contributed valuable insights into the trajectory of COVID-19 and served as a guide for policymakers to formulate responses that could lead to a manageable endemic state, ensuring public health resilience in the face of this ongoing global health challenge.

While our model represented an improvement over previous research, there remained room for further refinement. For example, the rapid mutation rate of viruses, leading to the emergence of new virus variants and reinfection, demanding the model more accurately mirror the real-time complexities of the pandemic. Future research should explicitly incorporate the emergence of new variants with potentially altered transmission characteristics and immune escape capabilities into the model. Additionally, the model needs adapt to the fact that recovered individuals can become susceptible again. Such improvements would enable the model to provide more precise simulations and predictions of transition dynamics of COVID-19, which are crucial for crafting effective and timely public health responses to ongoing and future outbreaks.

The evolution of the virus remains unpredictable. The transition dynamics of COVID-19, influenced by factors such as virus variant evolution, the emergence of new variants, vaccination, and the unknown aspects of natural immunity, are complex and uncertain. There is no straightforward path from a pandemic to an endemic state. As editorial of “nature” pointed out “there won’t be a single ‘exit’ wave to mark the lifting of pandemic restrictions. Further waves of infection and death are likely to follow, either from new variants that arise in the population, or from variants imported as the country opens its borders to visitors”^41^. The US has already experienced five major COVID-19 waves and may face more. However, the encouraging news is that COVID-19 in the US is progressively approaching an endemic stage.

### Supplementary Information

Supplementary Information.

## Data Availability

The daily COVID-19 data was downloaded from https://github.com/nytimes/covid-19-data. The vaccination data, including the number of vaccines distributed, the number of people who received at least one shot of vaccinations and full vaccination for each state were downloaded from https://ourworldindata.org/us-states-vaccinations. Tao Xu, Xiaoxi Hu and Zixin Hu had full access to all of the data in the study and takes responsibility for the integrity of the data and the accuracy of the data analysis.
